# Robot‐assisted minimally invasive esophagectomy for esophageal cancer: Meticulous surgery minimizing postoperative complications

**DOI:** 10.1002/ags3.12390

**Published:** 2020-08-16

**Authors:** Kei Hosoda, Masahiro Niihara, Hiroki Harada, Keishi Yamashita, Naoki Hiki

**Affiliations:** ^1^ Department of Upper Gastrointestinal Surgery Kitasato University School of Medicine Sagamihara Japan; ^2^ Division of Advanced Surgical Oncology, Research and Development Center for New Medical Frontiers Kitasato University School of Medicine Sagamihara Japan

**Keywords:** complication, esophagus, minimally invasive esophagectomy, RAMIE, robotic surgery

## Abstract

Minimally invasive esophagectomy (MIE) has been reported to reduce postoperative complications especially pulmonary complications and have equivalent long‐term survival outcomes as compared to open esophagectomy. Robot‐assisted minimally invasive esophagectomy (RAMIE) using da Vinci surgical system (Intuitive Surgical, Sunnyvale, USA) is rapidly gaining attention because it helps surgeons to perform meticulous surgical procedures. McKeown RAMIE has been preferably performed in East Asia where squamous cell carcinoma which lies in more proximal esophagus than adenocarcinoma is a predominant histological type of esophageal cancer. On the other hand, Ivor Lewis RAMIE has been preferably performed in the Western countries where adenocarcinoma including Barrett esophageal cancer is the most frequent histology. Average rates of postoperative complications have been reported to be lower in Ivor Lewis RAMIE than those in McKeown RAMIE. Ivor Lewis RAMIE may get more attention for thoracic esophageal cancer. The studies comparing RAMIE and MIE where recurrent nerve lymphadenectomy was thoroughly performed reported that the rate of recurrent nerve injury is lower in RAMIE than in MIE. Recurrent nerve injury leads to serious complications such as aspiration pneumonia. It seems highly probable that RAMIE is beneficial in performing recurrent nerve lymphadenectomy. Surgery for esophageal cancer will probably be more centralized in hospitals with surgical robots, which enable accurate lymph node dissection with less complications, leading to improved outcomes for patients with esophageal cancer. RAMIE might occupy an important position in surgery for esophageal cancer.

## Graphical abstract

Robot‐assisted minimally invasive esophagectomy (RAMIE) is rapidly gaining attention. RAMIE has been reported to lead to lower rates of recurrent nerve injury than conventional minimally invasive esophagectomy, when recurrent nerve lymphadenectomy was thoroughly performed. RAMIE might occupy an important position in surgery for esophageal cancer.

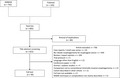

## 1 INTRODUCTION

Worldwide, 445 800 new esophageal cancer cases occurred, while 400 200 deaths occurred in 2012.^1^ Curative treatment for intrathoracic esophageal cancer comprises preoperative chemotherapy^2^, ^3^ or chemoradiotherapy^4^, ^5^ followed by surgical resection, which is the most invasive procedure in gastroenterological surgery resulting in 40% of the morbidity rate with a mortality rate of 3%, according to the National Clinical Database in Japan.^6^

Subtotal esophagectomy with extensive mediastinal lymphadenectomy remains a critical element in the treatment of esophageal cancer. Minimally invasive esophagectomy (MIE), which uses thoracoscope or laparoscope to minimize the surgical trauma to the thoracic or abdominal wall, has been introduced to reduce the operative stress in the area of esophageal surgery especially in high‐volume centers. Randomized controlled trials and meta‐analyses have revealed that MIE reduces postoperative complications, especially pulmonary complications, and has equivalent long‐term survival outcomes as compared to open esophagectomy.^7^, ^8^, ^9^ However, traditional thoracoscopic esophagectomy requires such high skill that only limited expert surgeons can perform this surgery. Some of the reasons that make this surgery so difficult are: limited range of movement of the instrument tip caused by narrow intercostal space; proximity of important organs such as trachea, main bronchi, and thoracic aorta, lymph node dissection around the recurrent nerves; and narrow upper mediastinum surgical space.

In 2000, da Vinci was approved in the Food and Drug Administration (FDA) as the first computerized telesurgical device in the United States.^10^ Initially, robot‐assisted surgery was widespread in the field of pelvic surgery, including prostate surgery and gynecological surgery. The da Vinci surgical system provides surgeons with a three‐dimensional camera, instruments with 7° freedom of movement, tremor filtration, and motion scaling, which enable surgeons to overcome the difficulty encountered in conventional MIE and to perform extremely delicate procedures needed for esophageal cancer surgery more easily and precisely.

In this article, we aim to highlight the development and current status of robot‐assisted minimally invasive esophagectomy (RAMIE) and compare it with conventional MIE, reviewing the pertinent literature.

## 2 STUDY SELECTION

**FIGURE 1:**
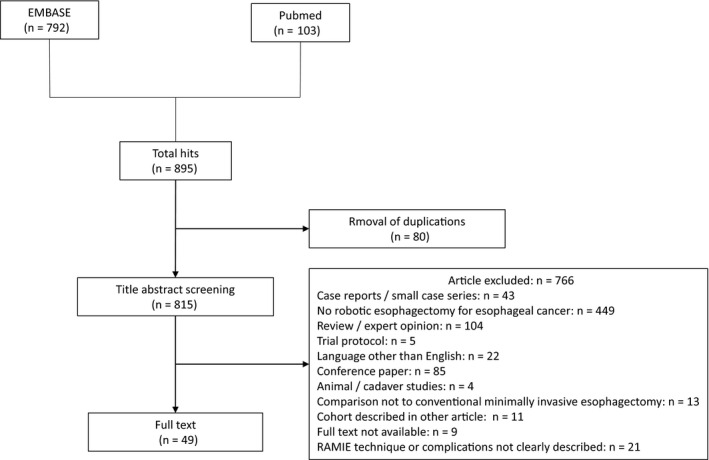
Flow chart of selection for included studies

A manual search using PubMed and Embase was conducted for references related to studies on RAMIE published until 30 March 2020. The following search terms were used: “Esophagus” and “robot.” A total of 49 out of 815 studies were selected that: (a) included more than 10 patients; (b) in which the RAMIE technique used was clearly described; and (c) in which the complications were adequately described. For manuscripts from the same institution, new reports were adopted if they were considered to contain the same cases (Figure 1).

## 3 CLASSIFICATION OF RAMIE

RAMIE is thought to be classified into three categories; transthoracic thoracoscopic esophagectomy with cervical anastomosis (McKeown RAMIE), transthoracic thoracoscopic esophagectomy with intrathoracic anastomosis (Ivor Lewis RAMIE), and transhiatal esophagectomy (transhiatal RAMIE).

## 4 MCKEOWN RAMIE

In the transthoracic RAMIE, McKeown procedure has been preferably performed for the ease of reconstruction, especially for middle or upper‐third thoracic esophageal cancer. The case of McKeown RAMIE with three‐field procedure was first reported by Kernstine and colleagues in 2004.^11^ The patient had T3N0 adenocarcinoma in the lower thoracic esophagus area and had undergone preoperative chemoradiotherapy of paclitaxel, carboplatin, and 40 Gy of radiation therapy. Robotic surgery was applied for both the thoracic and the abdominal procedures with the total surgical console time of 260 minutes and estimated blood loss (EBL) of 900 mL. They concluded that transthoracic RAMIE could potentially provide an oncologically superior resection with reduction of the burden to the patient. After this report, McKeown RAMIE has been favorably performed especially in East Asia where squamous cell carcinoma, which lies in more proximal esophagus than adenocarcinoma, is a predominant histological type of esophageal cancer.^12^, ^13^, ^14^

Table 1 lists case series studies of McKeown RAMIE including 10 cases or more. ^12^, ^13^, ^15^, ^16^, ^17^, ^18^, ^19^, ^20^, ^21^, ^22^, ^23^, ^24^, ^25^, ^26^ In most studies, squamous cell carcinoma was the predominant histological type. Median or average EBL was 100 mL or more in all studies reporting EBL with maximum median EBL of 950 mL. Median or average numbers of harvested lymph nodes were more than 20 in nine of the 13 studies. The average rates of postoperative complications were as follows: pneumonia 19.6%, anastomotic leak 15.1%, chyle leak 9.2%, recurrent nerve injury 15.9%. The average mortality rate was 4.0%.

**TABLE 1:** Case series of McKeown RAMIE

Author	Year	n	Country	Pathology (%)			Op time	EBL	HLN	Complications (%)				
				SCC	Adeno	Other				Pneumonia	Anast leak	Chyle leak	RN injury	Mortality^a^
van Hillegersberg^19^	2006	21	Netherland	52	48	0	450	950	20	48	14	14	14	5.0
Kernstine^20^	2007	14	USA	29	57	14	672	275	18^b^	21	14	7.0	14	7.1
Boone^21^	2009	47	Netherland	38	62	0	450	625	29	45	21	13	19	6.4
Kim^12^	2010	21	Korea	95	5	0	410b	150	40	0	19	4.8	29	0
Puntambekar^22^	2011	32	India	NA	NA	NA	210	NA	NA	6.3	9.3	9.3	6.2	NA
Kim^13^	2014	40	Korea	100	0	0	429^b^	158^b^	43^b^	13	10	5.0	20	2.5
van der Sluis^23^	2015	108	Netherland	19	72	9	381	340	26	33	19	18	9.3	5.0
Park^24^	2016	114	Korea	96	4	0	420^b^	209^b^	44^b^	9.6	15	1.8	26	2.6
Chiu^25^	2017	20	Hong Kong	85	15	0	500^b^	356^b^	18^b^	5	15	5.0	25	5.0
Somashekhar^26^	2017	35	India	74	26	0	NA	NA	32	2.9	0	0	2.9	0
van der Horst^15^	2017	31	Netherland	61	39	0	435	350	22	32	19	29	13	13
Goel^16^	2018	27	India	96	4	0	343^b^	208^b^	18^b^	7.4	11	3.7	7.4	3.7
Zhu^17^	2019	10	China	100	0	0	374	100	22	NA	NA	0	20	0
Hosoda^18^	2020	20	Japan	90	10	0	490	151	44	10	25	5.0	10	0
Average										19.6	15.1	9.2	15.9	4.0

Abbreviations: Adeno, Adenocarcinoma; Anast leak, Anastomotic leak; EBL, Estimated blood loss; HLN, Hevested lymph nodes in total; Op time, Operative time; RN injury, Recurrent nerve injury; SCC, Squamous cell carcinoma.

^a^ In hospital or 90‐d mortality.

^b^ Average otherwise median.

We started McKeown RAMIE in November 2018 and experienced 20 cases until January 2020. The number of 20 cases might not have completed the learning curve period. However, the short‐term outcomes were comparable to those in the other studies of McKeown RAMIE with median operative time of 490 minutes, median EBL of 151 mL, and median number of harvested recurrent nerve lymph nodes of 7.4. The rate of postoperative complications was also acceptable with pneumonia of 10% and recurrent nerve injury of 10%. RAMIE certainly offers advantages over MIE even in the introductory period.^18^

In 2019, Utrecht group reported the results of the first randomized controlled trial comparing McKeown RAMIE and open transthoracic esophagectomy (ROBOT trial).^27^ In this study, they compared 54 patients allocated to RAMIE and 55 patients allocated to open transthoracic esophagectomy and reported that RAMIE was better than open transthoracic esophagectomy in postoperative complication rate (59% vs 80%, *P* = .02) and functional recovery at postoperative day 14. The long‐term oncological outcome was comparable with each other. This trial provided evidence for the use of McKeown RAMIE to improve short‐term postoperative outcomes.

## 5 IVOR LEWIS RAMIE

On the other hand, Ivor Lewis RAMIE, which was first reported by Melvin et al in 2002,^10^ has been preferably performed in the United States where the adenocarcinoma, including Barrett esophageal cancer, is the most frequent histology in patients with esophageal cancer. Ivor Lewis esophagectomy requires intrathoracic anastomosis, which is relatively difficult, either by hand‐sewn or mechanical anastomosis when performed thoracoscopically. Surgical robot may help surgeons perform intrathoracic hand‐sewn anastomosis more proficiently as compared with that in conventional thoracoscopic MIE.

Recurrent nerve injury is reported to be significantly less in Ivor Lewis MIE than in McKeown MIE.^28^, ^29^, ^30^ Table 2 shows case series studies of Ivor Lewis RAMIE including more than 10 cases.^31^, ^32^, ^33^, ^34^, ^35^, ^36^, ^37^, ^38^, ^39^, ^40^, ^41^, ^42^ Only four studies reported recurrent nerve injury with the average rate of 4.2%.^31^, ^33^, ^37^, ^39^ On the other hand, recurrent nerve injury rates in the case series of McKeown RAMIE were reported to be 2.9%‐29% with the average rate of 15.9%.^12^, ^13^, ^15^, ^16^, ^17^, ^18^, ^19^, ^20^, ^21^, ^22^, ^23^, ^24^, ^25^, ^26^ However, it should be considered that McKeown and Ivor Lewis procedures have different operative indications and the attitudes toward upper mediastinal dissection would be different, which could affect the rate of recurrent nerve palsy and other complications.

**TABLE 2:** Case series of Ivor‐Lewis RAMIE

Author	Year	n	Country	Pathology (%)			Op time	EBL	HLN	Complications (%)				
				SCC	Adeno	Other				Pneumonia	RN injury	Anast leak	Chyle leak	Mortality^a^
Cerfolio^39^	2013	16	USA	19	81	0	367^b^	60^b^	18^b^	0	0	0	0	0
de la Fuente^38^	2013	50	USA	6	92	2	445	146	20	10	NA	2.0	4.0	0
Hernandez^34^	2013	52	USA	6	88	6	442	NA	19	9.6	NA	1.9	3.8	0
Trugeda^37^	2014	14	Spain	36	64	0	222^b^	75^b^	18^b^	0	0	7.1	14	0
Hodari^36^	2015	54	USA	6	85	9	362	74	16	14	NA	5.5	2.3	1.9
Wee^35^	2016	20	USA	10	75	15	455^b^	275^b^	23	10	NA	0	10	0
Egberts^32^	2017	75	Germany	0	96	4	392^b^	172^b^	29	NA	NA	9.6	NA	3.9
Zhang^31^	2018	61	China	95	0	5	316	189	19	6.6	8.2	9.8	1.6	0
Meredith^40^	2018	147	USA	10	86	4	415	158	20	6.8	NA	2.7	3.4	1.4
Pötscher^41^	2019	11	Austria	NA	NA	NA	389^b^	NA	NA	NA	NA	18	NA	NA
Wang^42^	2019	31	China	71	26	3	387	110^b^	17	3.2	NA	6.5	NA	0
var der Sluis^33^	2020	100	Germany	19	79	2	415	311	29	12	3.0	8.0	4.0	3.0
Average										8.5	4.2	5.6	3.7	1.5

Abbreviations: Adeno, Adenocarcinoma; Anast leak, Anastomotic leak; EBL, Estimated blood loss; HLN, Hevested lymph nodes in total; Op time, Operative time; RN injury, Recurrent nerve injury; SCC, Squamous cell carcinoma.

^a^ In hospital or 90‐d mortality.

^b^ Median otherwise average.

In most studies, adenocarcinoma was the predominant histological type. Median or average EBL was <100 mL in three of the 10 studies reporting EBL with maximum average EBL of 311 mL. Median or average numbers of harvested lymph nodes were more than 20 in only three of the 11 studies. The average rates of postoperative complications were lower than McKeown RAMIE, as follows: pneumonia 8.5%; anastomotic leak 5.6%; chyle leak 3.7%; recurrent nerve injury 4.2%. The average mortality rate was 1.5% and was also lower than McKeown RAMIE. For the patients in whom the rate of lymph node metastasis in the superior mediastinum is suspected to be very low, or when the advanced MIE (RAMIE) can realize radical superior mediastinal lymph node dissection easily, Ivor Lewis RAMIE may get more attention for thoracic esophageal cancer.

## 6 TRANSHIATAL RAMIE

Another robotic esophagectomy, transhiatal RAMIE, was first reported by Horgan et al in 2003.^43^ One of the greatest advantages of this operation is that it can be performed without separate pulmonary ventilation or artificial pneumothorax, which enables esophagectomy even in patients with possible intrathoracic adhesion or low respiratory function.

Transhiatal RAMIE in combination with transcervical upper mediastinal dissection is quite different from transhiatal RAMIE in Western countries.^44^ Mori et al claimed that surgical robot with articulating instrument enables the same extent of lymph node dissection as that achieved in open transthoracic esophagectomy. It has been reported that transhiatal RAMIE had less postoperative pneumonia with improved postoperative quality of life than open transthoracic esophagectomy.^44^, ^45^ Although this surgery has a possibility to become widespread, mediastinoscopic anatomical knowledge is not widely known, which may lead to life‐threatening organ damage such as damage of bronchi which lie at the deepest point from both the abdomen and the neck.

Robotic transcervical recurrent nerve lymph node dissection in combination with transhiatal RAMIE using da Vinci Xi has recently been reported as an innovative treatment.^46^, ^47^ This transcervical method enables recurrent nerve lymph node dissection by robotic approach. The rate of recurrent nerve injury is currently reported to be relatively high (25%‐33%),^46^, ^47^ but this might be because of an incomplete learning curve. More recently, though using a cadaveric model, a preclinical study demonstrated that transcervical esophagectomy is technically feasible with the novel da Vinci SP Surgical System without additional ports or assistance.^48^ In this cadaveric study, all the thoracic procedures including the dissection of subcarinal lymph nodes and lower mediastinal lymph nodes were successfully performed from the neck. Although clinical trials are needed to prove the feasibility in clinical setting, this transcervical RAMIE might become the ultimate minimally invasive esophagectomy with radical mediastinal lymph node dissection for esophageal cancer.

## 7 COMPARISON OF SHORT‐TERM OUTCOMES BETWEEN RAMIE AND MIE

One systematic review with meta‐analysis of retrospective studies comparing short‐term outcomes between RAMIE and MIE has been reported.^49^ However, no prospective randomized controlled trial comparing RAMIE and MIE has been reported yet. Table 3 shows 12 retrospective studies comparing short‐term outcomes of RAMIE and MIE.^14^, ^50^, ^51^, ^52^, ^53^, ^54^, ^55^, ^56^, ^57^, ^58^, ^59^, ^60^ In seven of these studies, propensity score matching was conducted to balance confounding factors to reduce possible bias.

**TABLE 3:** Comparison of short‐term outcomes between RAMIE and MIE in previous retrospective studies

Author	Year	Country	Matching	Method	n	Op time	EBL	HLN		Complications (%)				
								Total	Thoracic	Pneumonia	RN injury	Anast leak	Chyle leak	Mortality^c^
Suda^14^	2012	Japan	Non	RAMIE	16	682^a^	144^a^	37^a^	18^a^	6.0	38	38	0	0
				MIE	20	649^a^	139^a^	39^a^	22^a^	20	75	10	10	0
Weksler^50^	2012	USA	Non	RAMIE	11	439	200	23	NA	9.1	9.1	9.1	NA	0
				MIE	26	484	226	23	NA	15	3.8	15	NA	7.6
Park^53^	2016	Korea	Non	RAMIE	62	490	463	37	NA	15	13	8.1	NA	1.6
				MIE	43	458	467	29	NA	14	24	2.3	NA	0
He^54^	2018	China	PS	RAMIE	27	349	119	20	NA	19	15	11	0	0
				MIE	27	285	158	19	NA	7.4	11	3.7	3.7	3.7
Chao^55^	2018	Taiwan	PS	RAMIE	34	231^b^	92	37	18	5.9	21	0	NA	0
				MIE	34	200^b^	103	36	19	18	29	5.9	NA	2.9
Deng^56^	2019	China	PS	RAMIE	52	353	96	22	12	9.6	14	5.8	0	3.8
				MIE	52	274	128	17	10	7.7	7.7	3.8	1.9	3.8
Tagkalos^57^	2019	Germany	PS	RAMIE	40	388^a^	339	27^a^	NA	15	NA	13	NA	5.0
				MIE	40	321^a^	343	23^a^	NA	18	NA	13	NA	2.5
Zhang^58^	2019	China	PS	RAMIE	66	302	200^a^	19	10	6.1	6.1	7.6	0	1.5
				MIE	66	275	200^a^	19	12	7.6	4.5	4.5	1.5	1.5
Chen^59^	2019	China	PS	RAMIE	54	187	119	25	NA	15	13	9.3	1.9	NA
				MIE	54	193	117	25	NA	24	32	3.7	3.7	NA
Motoyama^60^	2019	Japan	Non	RAMIE	21	634^a^	492^a^	52^a^	23^a^	0	24^c^	5.0	5.0	NA
				MIE	28	598^a^	385^a^	59^a^	20^a^	0	47^c^	8.0	3.0	NA
Yang^51^	2019	China	PS	RAMIE	271	244	211	20	12	8.9	29	12	1.5	NA
				MIE	271	276	210	19	12	13	15	14	0.7	0.7
Harbison^52^	2019	USA	Non	RAMIE	100	445	NA	NA	NA	11	NA	14	NA	3.0
				MIE	625	418	NA	NA	NA	19	NA	15	NA	2.2

Abbreviations: Anast leak, Anastomotic leak; EBL, Estimated blood loss; HLN, Hevested lymph nodes; Op time, Operative time; PS, propensity score matching; RN injury, Recurrent nerve injury.

^a^ Median otherwise average.

^b^ Thoracic operative time.

^c^ Left recurrent nerve injury.

^d^ In‐hospital or 90‐d mortality.

Most of the studies reported that operative time was longer in RAMIE. Robotic surgery includes docking and undocking of the patient cart, replacement of the instruments, etc. In this respect, robotic surgery tended to take longer time than conventional minimally invasive surgery. Six studies reported that EBL was lower in RAMIE than MIE, while five did not. The only systematic review with meta‐analysis reported that EBL was significantly lower in RAMIE than MIE. However, in a report by Yang et al, who analyzed the largest number of cases in RAMIE and MIE using propensity score matching, EBL was similar between the groups.^51^ If the learning curve of robotic surgery was completed, operative time would become shorter and EBL would become lower in RAMIE than in conventional MIE because of improved dexterity of the instruments.

In terms of lymph node dissection, six studies reported that RAMIE yielded a higher number of total harvested lymph nodes, whereas two reported that RAMIE yielded a lower number of total harvested lymph nodes. The number of harvested lymph nodes is recognized as one of the markers of surgical quality. The tendency of RAMIE to harvest a larger number of lymph nodes might indicate the higher surgical quality of RAMIE.

Regarding postoperative complications, pneumonia is an important life‐threatening complication with the incidence rate of postoperative pneumonia reported in almost all studies. None of these studies showed significant difference in the incidence rate of postoperative pneumonia. However, two studies which included 100 or more cases undergoing RAMIE reported a relatively lower rate of postoperative pneumonia.^51^, ^52^ On the other hand, no consistent trend was found regarding another important life‐threatening complication of anastomotic leak. Anastomotic methods for RAMIE and MIE were similar within the same study, though varied from study to study. RAMIE did not affect the anastomotic leakage rate.

## 8 DISSECTION OF RECURRENT NERVE LYMPH NODES

The extent and quality of recurrent nerve lymph node dissection substantially vary among different regions. In the Western countries, where predominant histological type of esophageal cancer is adenocarcinoma and preoperative chemoradiotherapy followed by surgical resection is the standard of care for esophageal cancer, recurrent nerve lymph node dissection may be quite different from that performed in Japan. Therefore, comparison of recurrent nerve injury rate between the studies does not directly translate into the comparison of the quality of esophageal surgery. There have been four studies which compared RAMIE and MIE where the methods of recurrent nerve dissection were described in detail and the number of harvested recurrent nerve lymph nodes was five or more.^14^, ^55^, ^60^, ^61^ All these studies reported that the rate of recurrent nerve injury is lower in RAMIE than in MIE. Recurrent laryngeal nerve injury leads to serious complications such as aspiration pneumonia. It is highly probable that RAMIE is beneficial in performing extended upper mediastinal lymph node dissection.

One of the keys to successful lymph node dissection around the recurrent nerves is how to avoid tractional damage to the nerves. The surgical robot can help surgeons perform the lymph node dissection with minimum tractional damage with intuitive and meticulous manipulations. Hiki demonstrated that minimally invasiveness in laparoscopic gastrectomy derives from less manipulation, such as intestinal manipulation and pancreatic compression, which he calls “organ‐touchless surgery.”^62^ We think that this concept is in line with that in the successful recurrent nerve lymph node dissection in RAMIE.

## 9 FUTURE DIRECTION

In April 2018, robot‐assisted thoracoscopic esophagectomy was covered by the national insurance in Japan. Subsequently, robot‐assisted mediastinoscopic esophagectomy was also covered by the national insurance in Japan in April 2020. Japan has been far behind Europe, the US, Korea, and China in the field of robotic surgery partly because RAMIE had not been covered by medical insurance until recently and Japanese national insurance prohibits mixed medical care. Therefore, once a patient hopes to undergo RAMIE, he or she had to pay the large medical expenses related to the disease without insurance. However, due to the potential benefit provided by the surgical robot, RAMIE is rapidly prevailing, especially in advanced medical institutes in Japan. Compared with conventional MIE, the maneuverability is improved, the physical burden on the operator is reduced. The significance of robot‐assisted thoracoscopic esophagectomy seems overwhelming.

On the other hand, there are numerous reports showing that surgical outcomes, including postoperative morbidity and mortality, were better in high‐volume centers than those in low‐volume centers.^63^, ^64^, ^65^ Though not as many as in other countries, surgical robots have been installed in the majority of leading high‐volume centers in Japan but might not be installed in many other centers. This may be because only high‐volume centers can afford to buy and maintain running these robots, as there is a large financial burden associated with the da Vinci surgical system. Surgery for esophageal cancer will probably be more centralized in hospitals with surgical robots, leading to improved outcomes for esophageal cancer surgery.

There are two ongoing multicenter prospective randomized controlled trials comparing RAMIE and MIE which are called “REVATE” trial^66^ and “RAMIE” trial.^67^ “REVATE” trial is designed to demonstrate the superiority of RAMIE regarding recurrent nerve lymph node dissection. The primary endpoint is set to be the rate of unsuccessful recurrent nerve lymph node dissection defined as failure to remove lymph nodes along the left recurrent nerve or occurrence of permanent left recurrent nerve injury. “RAMIE” trial is designed to demonstrate non‐inferiority of RAMIE in overall survival. These trials will provide important evidence of usefulness of RAMIE compared to MIE.

## 10 CONCLUSION

RAMIE is one of the operations that can maximize the advantages of surgical robots. Most of the studies reported so far dealt with the initial experience of RAMIE. According to these results, the safety and feasibility of RAMIE during the learning period were confirmed. In the surgical resection for esophageal squamous carcinoma, which is a predominant histological type of esophageal cancer in East Asia including Japan, lymph node dissection around recurrent nerve is the most important point. This recurrent nerve lymph node dissection is where the robotic surgery can be most beneficial through precise movement of robotic instrument. Esophageal cancer surgery including RAMIE will be centralized more and more. Although the entire field of RAMIE is still so immature that further studies are needed to demonstrate the superiority of RAMIE to the other surgical methods, RAMIE might occupy an important position in surgery for esophageal cancer.

## CONFLICT OF INTERESTS

Authors declare no conflict of interests for this article.
